# Feasibility and acceptability outcomes of the InMe trial - a randomised controlled trial in participants with subclinical eating and somatic symptom disorders

**DOI:** 10.1371/journal.pone.0342307

**Published:** 2026-02-04

**Authors:** Marina Bobou, Michal Tanzer, Alkistis Saramandi, Caroline Selai, Aikaterini Fotopoulou

**Affiliations:** 1 Research Department of Clinical, Educational and Health Psychology, University College London, London, United Kingdom; 2 Department of Clinical and Movement Neurosciences, UCL Queen Square Institute of Neurology, London, United Kingdom; University of Turin, ITALY

## Abstract

Dysregulations in interoception have been associated with mental health disorders including eating and somatic symptom disorders. The present study addresses the feasibility and acceptability of a novel, behavioural intervention (InMe) in a healthy sample with low, self-reported interoception. The efficacy of the InMe intervention against an active control arm was tested in a randomised controlled trial (RCT) reported elsewhere, while the feasibility and acceptability of InMe were assessed in parallel and are fully reported here. Participants were randomly assigned to the intervention arm (InMe) or active control arm and stratified according to their self-reported gender and a cut-off score from the Eating Disorders Questionnaire (EDE-Q). Feasibility and accessibility measures included self-report scales and questionnaires, assessor checklists and ratings, as well as behavioural and physiological responses. Data was gathered from a total of 102 participants and encompassed trial recruitment and retention rates, the suitability of measurement tools, as well as the feasibility and acceptability of stressors, interventions and other trial procedures. The study found satisfactory feasibility in recruitment procedures, trial measurement tools, and intervention procedures. Participants perceived the intervention as acceptable, though minor adjustments for trial optimisation were identified. Overall, this feasibility study provided promising evidence regarding the acceptability and feasibility of an interoception based intervention in a RCT context. These findings offer valuable insights particularly for the design of future clinical trials for testing the efficacy of this intervention further in clinical populations.

## Introduction

Interoception is defined as the process of sensing, integrating, and interpreting body signals that arise internally, from major visceral systems including the cardiovascular, gastrointestinal, and respiratory [1]. Dysregulations in interoceptive signal processing have been associated with the onset and maintenance of mental health disorders [1–3] including eating disorders (EDs) [4–6] and somatic symptom disorders [7–9]. Additionally, poor interoception has been linked to symptoms shared across mental health diagnoses [1,2], including impairments in emotion processing and regulation [10,11], alexithymia [12] and cognitive deficits in decision making and reward processing [13]. Specifically, in the case of EDs, dysregulations in interoceptive processing have been linked to clinical symptoms, as patients often struggle to interpret visceral signals of satiety and hunger [5,14]. However, evidence shows that such difficulties extend beyond eating and into other domains, such as the cardiac domain [14,15], potentially pointing towards a shared impairment. Similarly, interoceptive deficits and aberrant appraisal of interoceptive signals [8,16] have been linked with somatic symptom disorders, which are characterised by excessive preoccupation and hypervigilance towards physical symptoms such as weakness and pain [17].

### Interventions on interoception

Given the observed associations between mental health symptoms and interoception, various interventions have been developed to enhance interoception in both healthy individuals and those with mental health disorders [18]. A range of psychological interventions, such as interoceptive interventions in the context of cognitive behavioural therapy (CBT), aim to support patients to change their beliefs about interoception by appraising their interoceptive signals in adaptive ways [19]. Additionally, other techniques, including guided slow breathing [20], physical exercise [21,22], neuromodulation [23] and pharmaceutical medication [24,25] have also gained prominence. Unlike psychological interventions, these techniques have distinct mechanisms of action that modulate the interoceptive signal itself, or its regulation. Interventions targeting both levels of interoception processing have been recognised as more efficacious in increasing interoception [1,26]. However, before these interventions can be introduced into clinical practice, research must first determine whether they are feasible and acceptable.

Few studies have assessed the feasibility and acceptability of interoception based interventions [27–31]. Feasibility studies have shown that such interventions can indeed be feasible and acceptable amongst participants with a variety of mental health symptoms, including chronic pain [31] and suicidal ideation [27]. However, evidence in clinical populations remains limited, as to our knowledge, there is no research on the acceptability and feasibility outcomes of an intervention targeting both levels of interoception in a randomised controlled setting. Furthermore, no feasibility or acceptability studies have been conducted in individuals with self-reported disordered eating and somatic symptoms. To address this gap in research and provide valuable insights, we developed the Interoceptive iNsight and Metacognitive Efficacy beliefs (InMe) randomised controlled trial (RCT), which evaluates the efficacy of a novel therapeutic intervention targeting both levels of interoception processing.

### Intervention background and scope

The InMe trial was developed based on an audit that our research team conducted, on an EDs ward at a National Health Service (NHS) facility [32].The audit revealed biofeedback as a valuable tool for interoceptive interventions, offering the potential to enhance standard practices in the treatment of EDs. Building on these promising findings, the InMe trial was designed to evaluate the effectiveness of a novel therapeutic intervention. This trial was conducted with a subclinical population of university students, with the results intended to guide future research on the intervention’s efficacy in an NHS setting with clinical populations. The aim was to explore whether cardiac biofeedback, slow breathing, and interoceptive belief training can significantly enhance participants’ beliefs about their body. The main results on the efficacy of the intervention are reported elsewhere [33]. In the current paper, we focus exclusively on the trial’s feasibility and acceptability prior to its introduction into clinical practice, integrating participants’ and researchers’ perspectives to inform its implementation. Accordingly, we present key methodological aspects to clarify the context in which the trial’s feasibility and acceptability were evaluated.

### Overview of trial procedures

Eligible participants were randomly allocated to the intervention condition (InMe) or the active control condition (i.e., guided imagery) and stratified according to eating disorder symptoms and gender. Participants attended three in-person sessions, including two intervention sessions scheduled between 6–8 days apart and a follow up session occurring 7–10 weeks after the second session. During the first two sessions, participants received psychoeducation on interoceptive regulation and were exposed to the Trier Social Stressor Test (TSST) [34,35]. They were then invited to practice their assigned calming technique according to their randomisation arm. Participants in the InMe arm practiced slow breathing using cardiac biofeedback, whereas those in the active control arm practiced the guided imagery technique. Both calming techniques were designed to reduce stress and decrease participants’ their heart rate.

### TSST procedures

Following psychoeducation, participants completed the TSST [35] to induce a stress response in both arms. The procedure involved a three minute preparation period for an interview to join a high status managerial position or a prestigious university society. Participants delivered a three minute speech in front of a panel while being video recorded. After the participants’ speech, the panel members left the room, and the experimenter instructed the participant to perform the calming technique they previously learned. The InMe arm involved the use of a smartwatch with real-time biofeedback on participants’ heart rate, so participants could understand their physiological responses. For the second stressor (i.e., a verbal mental arithmetic task), the panel members re-entered the room, and the participant was asked to perform a three minute arithmetic task, which was also video recorded. In the InMe arm, participants were able to use the biofeedback from the watch to learn about their heart rate response to the stressors and the calming technique.

### Cardiac biofeedback

Participants’ heart rate was monitored throughout each session and participants in the intervention arm could visually observe their heart rate. The Polar 2 Ignite watch was considered an appropriate choice, as it employs optical heart rate monitoring in real time in a non-invasive approach [36]. An optical heart rate monitor has an LED (light emitting diode) and a photodiode sensor, which measure light absorbance on the skin capillaries. As capillaries fill with blood following heart systole, light absorbance changes, and therefore the watch determines the frequency of heart beats, providing the heart rate [37–39]. Cardiac biofeedback, a technique of visualising heart rate changes under conditions of stress or relaxation, helps participants understand and control these fluctuations. In this intervention, cardiac biofeedback was used to train individuals in heart rate regulation following stress induction in the InMe arm.

### Slow breathing technique

A slow breathing practice app, provided by the watch, was employed for the purposes of the intervention to help participants slow down their heart rate following stress induction [40]. The Serene breathing practice app guided participants via visual and vibratory cues to perform a slow breathing exercise of 6 breaths per minute for three minutes, which aimed to slow their heart rate. The heart rate is affected by breathing due to respiratory sinus arrhythmia (RSA), where heart rate increases during inhalation and decreases during exhalation. This synchrony helps optimise pulmonary gas exchange and regulate blood flow across body systems. In particular, a breathing rhythm of six breaths per minute has been shown to enhance heart rate variability, which is the fluctuation between two consecutive heart beats [40–42]. Participants were therefore instructed to practice the slow breathing technique after stress induction in the InMe arm, allowing them to reduce their stress response and learn to regulate their heart rate.

### Guided Imagery technique

The guided imagery was the relaxation technique used for participants assigned to the active control condition. As above, participants had three minutes to follow guidance narrated by a member of the research team, to relax following stress induction. Guided imagery technique started as follows: “s*tart by finding a comfortable position with your legs uncrossed. Feel free to close your eyes or leave them with a soft gaze towards the ground.* *Imagine a place where you feel calm and peaceful and easy*…”. The full guided imagery technique script is available in the S1 Table.

### Patient and Public Involvement

The trial protocol and design, including the standard operating procedures and patient facing documents (consent form and information sheet), were developed with the input of collaborators, including members of the public, experts by experience, and a clinical and academic steering committee. These Patient and Public Involvement (PPI) efforts have been published separately [43].

### Aims

To our knowledge, this is the first study assessing the feasibility and acceptability outcomes of an RCT that targets interoception at a behavioural level and a physiological level (i.e., fluctuations of heart rate). Investigating feasibility and acceptability within a university population is key to understanding whether and how this intervention can be implemented in subclinical settings (e.g., universities), as well as how it can be further adapted and eventually translated into clinical settings, such as an EDs facility.

## Methods

### Ethical approval & consent

The RCT was considered and approved by the appropriate ethics committee at University College London (UCL), United Kingdom (CEHP/2019/577). All participants provided written, informed consent prior to taking part in the trial for detailed procedures, see the main results publication [44].

### Inclusion/ Exclusion criteria

Participants were recruited from social media platforms and the SONA systems online subject pool, supported by the Division of Psychology and Language Sciences, UCL. Participants were deemed eligible if they scored below the 30^th^ percentile (i.e., ≤ 67) on the Body Awareness Questionnaire (BAQ) [45] and were aged between 18 and 30 years. Participants with current self-reported substance dependency, heart conditions, severe cognitive impairment, and severe neurological (e.g., epilepsy) or mental health conditions (e.g., psychosis) were excluded. In addition, participants were excluded if they were currently pregnant, using medication that could influence blood pressure, had a BMI ≥ 30, had self-reported vision or hearing problems that could not be corrected, or had inadequate self-reported English literacy, as these factors might affect interoception or comprehension of trial procedures.

### Recruitment

**Fig 1:**
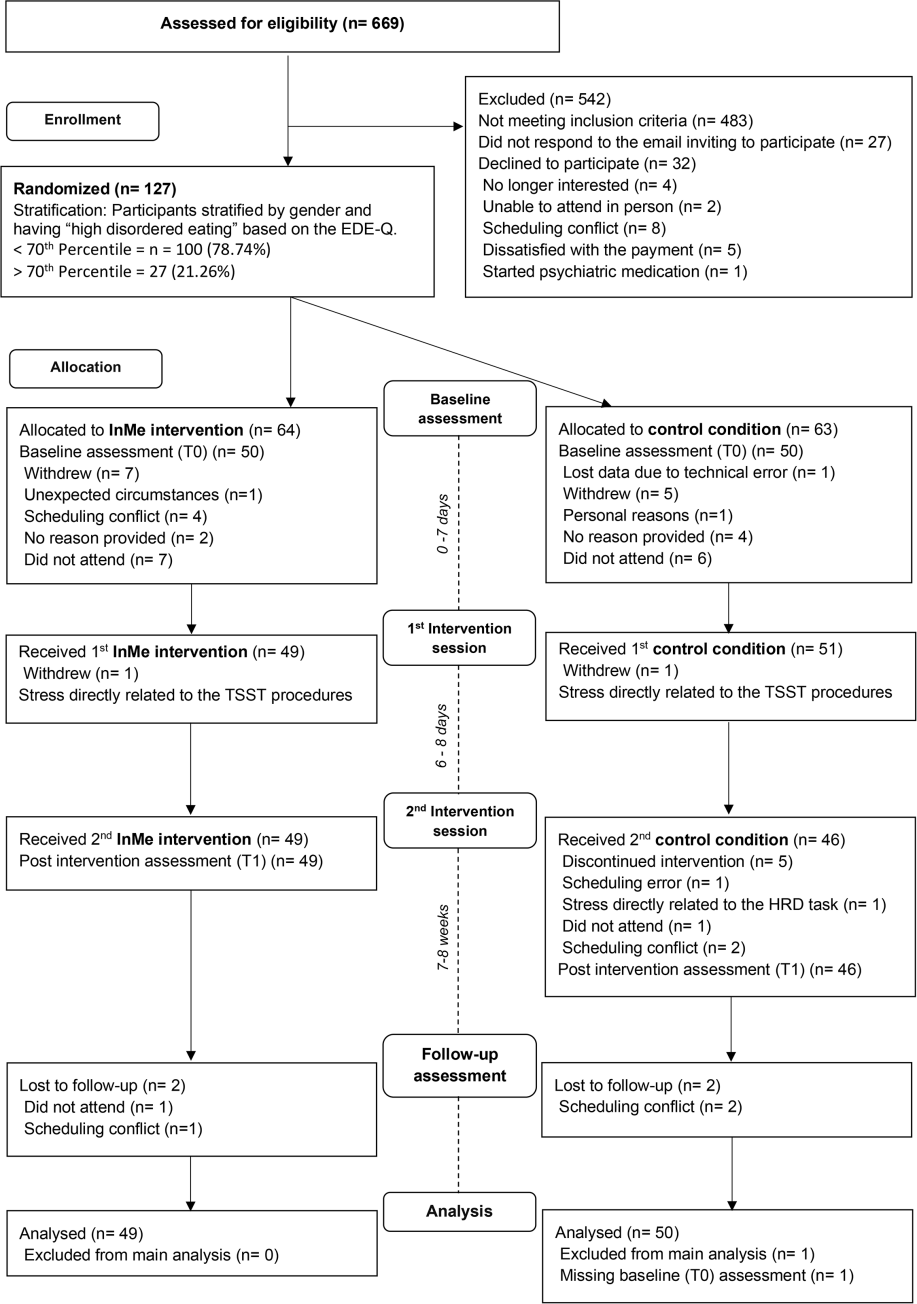
CONSORT flow diagram of participant recruitment, randomisation, allocation, and trial sessions.

The recruitment was reported in adherence to CONSORT guidelines, shown in Fig 1 below [46]. Given the association between disordered eating and interoception, eligible participants were stratified to either study arm according to disordered eating symptomatology, based on the global score of the Eating Disorder Examination-Questionnaire (EDE-Q) [47]. Individuals scoring at or above the 70^th^ percentile of the EDE-Q (2.33 for females and 1.48 for males) were assigned to the “high disordered eating” group, and conversely, individuals scoring below that percentile were classified in the “low disordered eating” group. Stratification allowed within study arm comparisons on participants’ attitudes and acceptability towards the intervention. Similarly, participants were stratified according to gender due to known differences in interoceptive accuracy [48]. With the above information in mind, the research team opted to recruit 120 participants between 01/07/2022 to 06/03/2023, which allowed approximately 10% participant attrition based on power analysis [33].

### Measures

#### Feasibility measures.

According to Eldridge et al., feasibility measures are necessary to inform whether a study can be done, should research proceed, and if so how [49]. Feasibility measures acquired did not evaluate the effect of therapy but rather included subjective, qualitative, and quantitative feedback from participants, observations from the research team, and objective measurements. Feasibility categories included testing procedures, researcher feedback, and intervention adherence. Data on trial testing procedures were necessary to acquire, as they would inform the planning of future interventions. The measures included recruitment and randomisation, which were reported according to the CONSORT guidelines [46].

Testing procedures included information on adverse event reporting and missing data. A separate adverse event protocol was developed to address potential adverse events occurring during the stressor procedures or the slow-breathing technique. The protocol specifically covered symptoms such as shortness of breath, light-headedness induced by the breathing exercise, or palpitations resulting from the stressors. As the trial had a complex design with different data modalities, acquired information on missingness of data and the occurrence of adversities helped inform whether elements of the trial should be removed. Trial questionnaire data were acquired electronically using the Qualtrics online platform. Participants were sent the baseline questionnaires online to complete prior to attending the first session, which included psychometric and demographic questionnaires. Additionally, participants were asked to complete online questionnaires throughout the in person testing sessions, which were administered by the experimenter. Missing questionnaire data during the testing session were attributed to researcher error or participant discontinuation of the intervention.

Researcher feedback feasibility measures constituted another important set of variables informing the practical aspects of the RCT, particularly with respect to session duration, staffing requirements, and testing facilities. This information was valuable for planning the trial and for understanding the practical resources and facilities required for its implementation.

Lastly, intervention specific feasibility measures were acquired on the adherence and efficacy of the stressors and the adherence to the slow breathing exercise. Stressor adherence was reflected by participants’ ability to perform all stressors during a testing session. To test the efficacy of stressors in changing participants’ heart rate, the following measurements were acquired. According to the preregistered study protocol, participants were classified as showing an inadequate stress response if they met either of two criteria: (1) a change of ± 2.5 standard deviations below the randomised arm mean across both stressors, or (2) a heart rate change of less than 5 heart beats before and after the stressor. To quantify these responses, heart rate measurements were acquired at pre-defined time points relevant to the experimental TSST procedure (e.g., before stressor 1). Criteria 1 and 2 measurements were calculated using data from the InMe group only, based on heart rate measurements at pre-defined time points (formulas shown in S2 Table). In addition, a third criterion was applied, which mirrored criterion 2 but used continuous, real time heart rate from the Polar watch during the stressor. Under criterion 3, a heart rate change of less than 5 heartbeats was also considered inadequate stress response. Unlike criteria 1 and 2, this criterion was based on heart rate data obtained from the wearable device and therefore applied to participants in both study arms. Participants who did not meet these criteria were excluded as outliers from the main data analysis. Adherence to the breathing technique was measured by the researchers, completing a checklist while the participant was following the watch guidance. The checklist consisted of the following items: (1) Is the participant actually looking at the watch? (2) Can you hear or notice breathing sounds from the participant? (3) Is the participant attempting to engage with the experimenter? and (4) Did the participant reach the light blue colour? Researchers marked the above questions on a *Yes/ No/ Don’t know* scale.

### Acceptability measures

Acceptability under the Sekhon et al., framework is considered a multi-faceted construct that reflects distinct components of the intervention, including participants’ affective attitude, burden, perceived effectiveness, ethicality, intervention coherence, opportunity costs, and self-efficacy [50]. Here, we focus on three acceptability constructs namely affective attitude; how an individual feels about the intervention, burden; the perceived amount of effort that is required to participate in the intervention, and self-efficacy; the participants’ confidence that they can perform the behaviours required to participate in the intervention. Specifically, participants’ affective attitude was acquired via structured questions on watch accuracy and comfort at the end of the intervention. Following the stressors of the first session and second session, participants in the InMe group were asked to rate how accurate the watch feedback was, on a scale of 0–100, with 0 corresponding to “Not at all accurate” and 100 corresponding to “Extremely accurate”. Additionally, participants in the intervention arm rated the watch comfort on a 5-point Likert scale after both testing sessions.

Participants’ burden and self-efficacy were measured by structured and open-ended questions on stressor appropriateness, usefulness and future practice of the slow breathing technique accordingly. Specifically, participants were asked to rate their agreement on a 5-point Likert scale to the following statements:”*The stressor tasks were appropriate and proportional to the aims of the study*”, “*The slow breathing practice with HR feedback from the watch was useful in regulating my stress reactions*”, “*It was very difficult to do the slow breathing technique to regulate my stress*”, and “*The HR feedback from the watch we tried today was useful in giving me a better understanding of how to gain control over my bodily and mental responses*”. Additionally, data on the practice of the calming technique were collected both between groups and across testing sessions. Specifically, the intervention and control groups were compared to determine whether participants in the intervention group were more likely to perform the calming technique. Practice was tracked across sessions to assess whether repeated exposure to the intervention provided additional benefits.

Finally, at the end of session 1 and 2, participants were asked the following open-ended questions: “*How useful did you find being able to see your heart rate while getting stressed, or after you did the breathing exercise?*” and “*Can you give me an example from your life of a stressful event or experience where you wished you had this kind of feedback to help you downregulate your stress?*”. Participants were invited to respond to the above questions in whichever way they deemed appropriate and to provide examples. Their responses were then grouped into categories reflecting the usefulness and future use of the intervention.

### Analysis

Statistical analysis was performed using R version 4.4.0 [51]. A paired t-test was conducted to determine whether adherence to the slow breathing exercise differed significantly between sessions. Additionally, a t-test was conducted to determine whether there were significant differences in practice rates between the InMe and control arms.

## Results

### Feasibility of testing procedures

A total of 669 participants completed the online screening, of which 186 (27.8%) were considered eligible and invited to participate (CONSORT diagram; Fig 1). The data indicated that just over 50% of eligible participants agreed to take part in the trial, suggesting that recruitment from a university population was feasible. Recruitment was challenging during exams, as the trial required multiple site visits; therefore, timing should be carefully considered. Additionally, time slots outside the standardised working schedule should be provided, facilitating the students’ schedule. However, translation to clinical, outpatient, and inpatient units will also differ. Overall, 127 (68.3%) of eligible participants agreed to participate, and all participants agreed to be randomly assigned to the InMe intervention and active control arm (CONSORT diagram; Fig 1).

Of the 127 participants randomised, the majority scored low on disordered eating symptoms, and 21.3% (27/127) scored high on the EDE-Q. Detailed EDE-Q high and low categories, PHQ-15 categories, and self-reported gender across the intervention and active control arms in the first session, are available in S3 Table. Overall, 21 participants with high ED symptoms attended the first session and 20 attended the whole intervention, including follow up (20/91, 22.0%). A similar pattern was detected in participants reporting moderate to severe somatic symptoms (PHQ-15 total ≥ 10). 14/22 participants with self-reported high somatic symptoms attended the first session, one withdrew in the second session and one in the follow up session (12/91), totalling 13.2% of participants scoring high on the PHQ-15 at follow up. Withdrawal rates of participants reporting disordered eating behaviours and somatic symptoms were low, which indicates that once enrolled and already attending the first session of the study, few participants withdrew.

### Adverse events

Potential adverse events occurring during the testing sessions, were noted in accordance with Good Clinical Practice (GCP) safety reporting guidelines, to optimise data integrity and participant safety. The adverse event protocol was developed with the input of PPI collaborators [43]. Although no adverse events were reported, one participant felt unwell following the first study session, which resulted in their withdrawal from the study. Specifically, the participant experienced discomfort while performing the heart rate discrimination (HRD) task [52], which required participants to attend to their heart rate. Such an event should be taken into consideration, as focusing on bodily signals may be distressing for clinical populations. Additionally, no adverse events were reported during the TSST procedures, although many participants were reluctant to complete either stressor due to the clearly stress-inducing nature of the tasks. Such disruptions to the protocol should be considered in future research, for example by incorporating alternative methods to elevate heart rate (e.g., physical exercise) that still allow participants to engage with the calming technique. Although not classified as an adverse event, one participant experienced light-headedness during the slow breathing exercise. The research team documented the incident and encouraged the participant to return to their normal breathing rhythm. Such a reaction could be explained by the transition from a state of stress to being asked to adjust their breathing to a slower rhythm and has been previously observed in the literature [53]. The suggested slow breathing rhythm might have not been optimal for the participant and therefore had the opposite of the desired outcome.

### Missing data

All technical challenges experienced during testing sessions were noted on the participant spreadsheet, which was useful for improving procedures for researchers and ensuring a comprehensive dataset. Specifically, a few technical challenges were experienced, which led to missing data and in some cases, deviations from the standardised study procedures, which are explained in Table 1 below. Specifically, due to technical limitations in the first few weeks of the trial, heart rate variability (HRV) measurements could not be obtained from the HRD task, which resulted in many participants having missing HRV data in the first session, (completeness: 56.9%; 58/102) and second session (completeness: 62.1%; 59/95). This was due to technical difficulties in embedding HRV measurements within the HRD task. Due to time restrictions, the research team opted to obtain HRV measurements using an alternative heart monitoring smartwatch (Empatica E4 watch). In addition to the technical issues obtaining HRV measurements at the onset of the trial, experimenter error in omitting HRV measurements, led to missing data during the follow-up session.

**Table 1:** Data completeness across the three sessions in both the InMe and Control arms.

Session (=N)	HRD	HRV	Arm	S1	S2	Other measurements
1 (=102)	102	58	InMe	45	42	100
			Control	48	48	
2 (=95)	95	59	InMe	44	45	95
			Control	46	46	
3 (=91)	85	80	–	–	–	85
			–	–	–	

HR: heart rate; HRD: heart rate discrimination task; HRV: heart rate variability; S1: first stressor; S2: second stressor; Other measurements: include BMI, watch adherence to breathing technique and notes obtained from each participant’s case report form.

Data were fully complete for the first two sessions of the HRD task, but during the follow up session six participants did not complete the task (completeness: 93.4%; 85/91). The absence of data could possibly be attributed to time constraints, as participants had limited availability and did not want to attend the follow-up session in person. In addition, future planning should consider the online completion of questionnaires, thereby reducing time spent at the study site and maximising efficient data collection. The pulse oximeter sensor measuring participants’ heart rate for the HRD task was fit appropriately, and thus all heart rate measurements were accurate.

Regarding the smartwatch, first session measurements, twelve (completeness: 39/51, 76.5%) of control participants did not have continuous heart rate recordings, and three participants (completeness: 48/51, 94.1%) had no data for that day. Additionally, two InMe arm participants (completeness: 49/51, 96.1%) did not have continuous heart rate recordings in the first session. The lack of continuous heart rate recordings can be attributed to experimenter error by not fitting the smartwatch appropriately to the participants’ wrist, or potentially the participant loosening the watch themselves, resulting in motion artefacts. In the second session, all control arm participants had heart rate recordings using the Polar watch, but seven participants (completeness: 39/46, 83.0%) lacked continuous heart rate recordings. Additionally, in the InMe arm, seven participants (completeness: 42/49, 85.7%), did not have continuous data, Therefore, greater caution should be encouraged in the study procedures, in fitting participants’ watches and ensuring their comfort with its wear.

### Online questionnaires

One participant did not complete the questionnaires prior to attending, due to experimenter oversight, mistakenly confirming the baseline questionnaire completion prior to the testing session. Additionally, information on participants beliefs in performing the HRD task and TSST procedures was acquired. Overall, HRD task related beliefs were shown to have the most missing values during the first session 3.9% (4/102) and 8.7% at the follow up session (8/92). HRD related beliefs had more missing data primarily, as experimenters omitted additional questionnaires during the HRD task. Such errors could be minimised in future practice if researchers receive further training in implementing checklists and there is more supervision, or overall reduction of some measures. Additionally, stressor related belief data were missing across both testing sessions (session 1: 7/102; 6.9%; session 2: 7/95 7.4%), due to participants refusing to perform the TSST in front of the panel, rather than due to experimenter error. Researchers considered it appropriate to proceed with the TSST procedure, even if participants refused to preform one of the stressors, as it would still provide participants the opportunity to downregulate their heart rate using the assigned technique after one stressor. Overall, it is evident that data acquisition via online questionnaires was feasible, and the quantity of missing data was low, but improvements can be made to minimise such risks even further.

### Researchers perspective

#### Training and facilities.

The trial required two separate testing rooms, for the TSST procedures to be conducted appropriately. This did not pose an issue, given the UCL facilities availability, but it might not be feasible when planning similar stressor procedures, in a larger research setting. In addition, two further experimenters were present, acting as panel members for the TSST procedures. However, this was achievable, as in some cases there were less experimenters available for the procedures. Specifically, for session one InMe arm 72.0% (36/50) of the TSST procedures had three experimenters, for session one control arm 68.6% (35/51) of the stress procedures had three experimenters, whereas for session two InMe arm 61.2% (30/49) of the TSST procedures had three experimenters and for session two control arm 76.1% (35/46) of the TSST procedures had three experimenters, 19.6% (9/46) had two experimenters and 4.3% (2/46) had only one experimenter. Imbalance in experimental panel and non-standardised number of panel members during the TSST procedures could introduce bias, something to be taken into consideration and controlled for future planning. Experimenters were gradually trained on average four to five sessions, from shadowing testing sessions, and attending the TSST as panel members, to being able to lead participant testing sessions. To avoid the incident of only one researcher carrying out TSST procedures, testing slots should be carefully booked, allowing flexibility to researchers in the event of unexpected cancellations or bookings, to ensure efficient implementation.

### Testing duration

Session 1 and session 2 were the same duration, which was approximately 1 hour and 38 minutes, for both the control and InMe arms. Comparatively, the InMe arm sessions were longer than the control arm session, for approximately 15 minutes. Each follow up session lasted 1 hour, with no time differences being observed between the control and InMe arms (shown in S4 Table). Regarding online procedures, time prior to the study site, participants completed the consent, screening, and baseline questionnaires in 50 minutes on average. The online administration of pre-visit procedures and the length of the study was deemed feasible and was considered appropriate for the procedures and relevant data acquisition.

### Feasibility of intervention

#### Stressor adherence.

**Fig 2:**
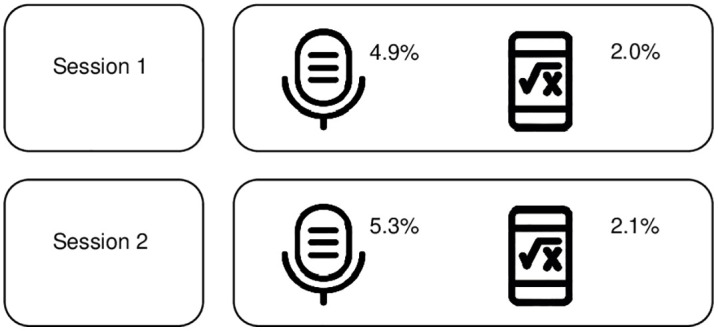
Visual representation of job, society and math stressor missing data across both sessions.

Participant adherence to the stressors and intervention was a key measure in the feasibility study, as it offered insights for potential procedural adjustments. The majority of participants proceeded with the stressor tasks. The percentage of participants not performing the stressors is shown in Fig 2 below. Participants performing either one of the stressors were not excluded from the experimental procedures, as they were still able to downregulate their heart rate, using the calming technique following one stressor. Only one participant refused to perform either of the stressors and was thus excluded from the trial. Fig 2 suggests that participants were more likely to engage in the math stressor than in the job or society interview stressors, as the latter of which may have been perceived to be more challenging.

### Stressor efficacy

**Fig 3:**
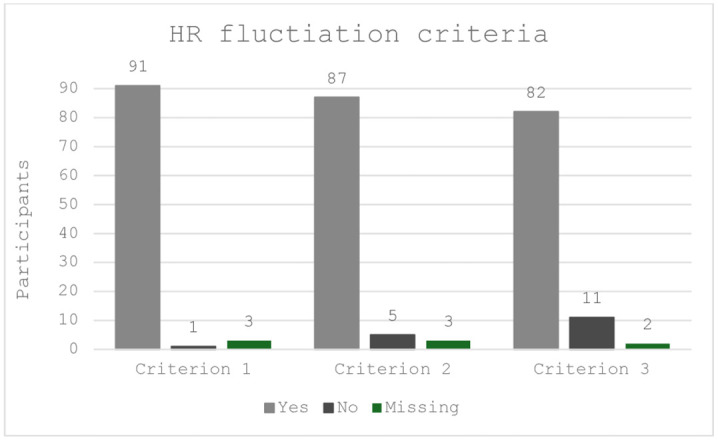
Graphical presentation of each criteria for HR upregulation following stress exposure and HR downregulations post calming technique, across both stressors in both session 1 and 2. Notes: Yes: Participants fulfilling the criterion, No: Participants not fulfilling the criterion and Missing: Participants with no data.

Efficacy of the stressors to elicit a heart rate response was assessed by three criteria as detailed in the methods section above. Participants not responding to any stressors were considered outliers and removed from subsequent analysis. Results are graphically summarised in Fig 3 below. Across the four stressors, it is evident that criterion 3 is more stringent, as fewer participants (11.6%) were able to fulfil it and is indeed the most accurate as it provides real time measurements with the least missing heart rate values. Additionally, participants with self-reported disordered eating and somatic symptoms were stressed during the TSST and able do downregulate their HR. The numbers do not differ significantly from the low disordered eating group as only two participants with high disordered eating symptoms did not fulfil criterion 2 and one did not fulfil criterion 3.

### Breathing adherence

**Fig 4:**
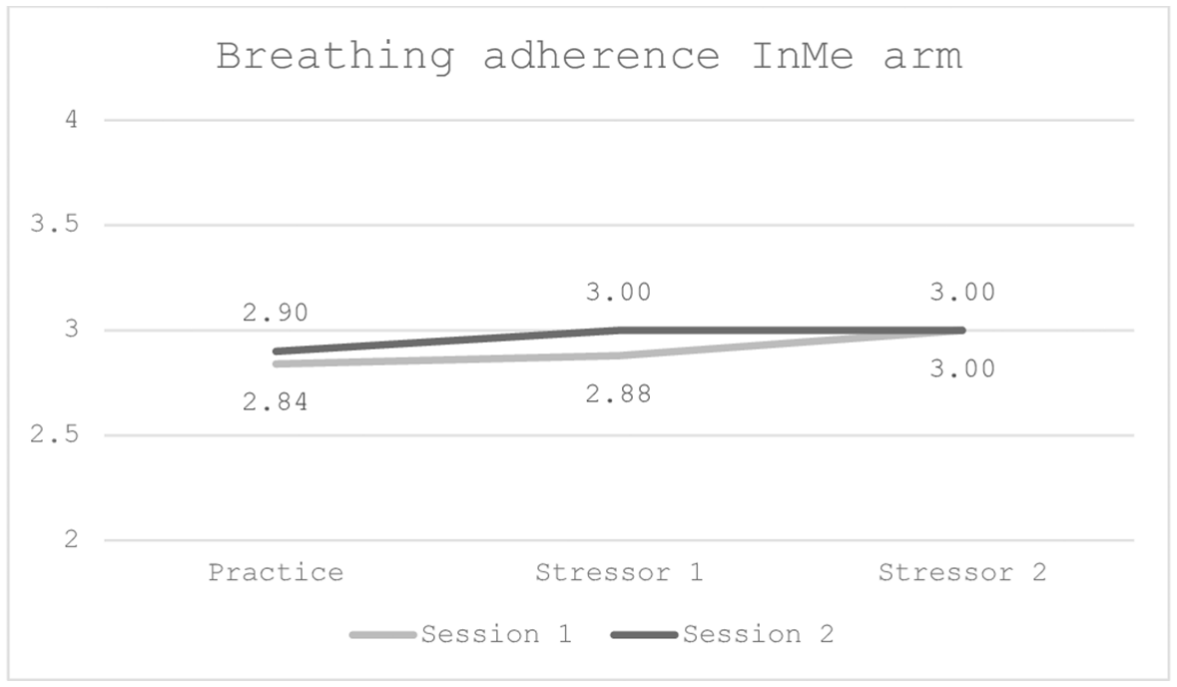
Graphic presentation of smartwatch breathing adherence in the InMe arm, across the two sessions.

The breathing technique was considered feasible, as adherence measured by the researchers remained high throughout the trial. Adherence did not reach maximum score (total = 4), but adherence continuously improved throughout the trial. A score of 3 was therefore considered adequate adherence to render the breathing technique feasible, as reaching the light blue zone on the checklist was challenging to fulfil. Paired t-test was performed, and no significant differences between the sessions were identified during practice (*t* = −0.31, *df* = 48, 95% CI [−0.60, 0.44], *p* = .76), following the first stressor (*t* = 0.15, *df* = 44, 95% CI [−0.55; 0.64], *p* = .88) and the second stressor (*t* = 0.21, *df* = 45, CI 95% [−0.56; 0.69], *p* = .84), as shown in Fig 4 below.

### Acceptability measures

#### Stressor appropriateness.

**Fig 5:**
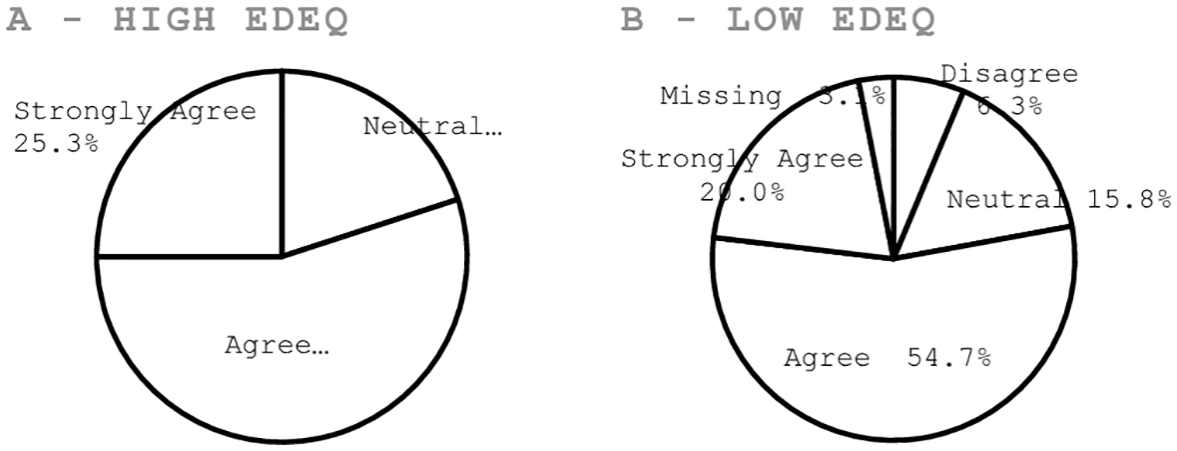
Participants’ responses on stressor appropriateness on a 5-point Likert scale, on the second testing session (n = 95). Panel A refers to the High EDE-Q sample; Panel B refers to the overall sample with a low EDE-Q score.

Participants were asked to rate the appropriateness of the presented stressors on a 5-point Likert scale. If the stressors were deemed disproportionately challenging, the research team would need to reconsider and adapt them to better suit the study population. The majority of participants c onsidered the stressors appropriate, which also aligned with the view of participants experiencing disordered eating symptoms, which is encouraging for stressor acceptability in a subclinical population, shown in Fig 5 below. Additionally, this feedback emphasises the limited drop-out rates associated with the stressor and the qualitative responses of participants regarding stressor appropriateness, and affirms the decision to use the TSST, which is widely regarded as the gold standard for acute stress induction in human participants [34,35].

### Acceptability of slow breathing and watch

#### Heart rate regulation.

Participants in the InMe group following the second session, were asked to provide their feedback on the slow breathing technique for the Serene app on the smartwatch. Feedback was provided on a 5-point Likert scale and is summarised in Table 2 below. A third of participants found the breathing technique challenging, which could be due to the limited practice sessions prior to the stressor exposure. Since participants considered the technique useful yet challenging, researchers should consider more time for participants to practice before stressor exposure. For example, alternative modes of practicing the slow-breathing technique could be implemented beyond watch based guidance, which some participants may find difficult to follow. In addition, piloting a range of breathing rhythms may allow participants to identify the pattern most suited to them, thereby facilitating more effective heart rate downregulation. This finding is encouraging for implementing the intervention on a larger scale, although the slow breathing practice may need to be personalised.

**Table 2:** Participant responses on a 5-point Likert scale on the slow breathing practice and HR feedback in the second testing session n = 95.

Statements	Strongly disagree	Disagree	Neutral	Agree	Strongly agree	Missing
The slow breathing practice with HR feedback from the watch was useful in regulating my stress reactions.	1.1%	3.2%	5.3%	28.4%	10.5%	51.6%
It was very difficult to do the slow breathing technique to regulate my stress.	6.3%	14.7%	13.7%	12.6%	1.1%	51.6%
The HR feedback from the watch we tried today was useful in giving me a better understanding of how to gain control over my bodily and mental responses.	0.0%	4.2%	3.2%	31.6%	9.5%	51.6%

### Watch accuracy & comfort

Participants reported a mean accuracy of above 60 across both sessions when their heart rate increased and when it decreased, shown in Table 3 below. Similarly, after the second session, most participants considered the watch comfortable to wear, which provides insights on whether the watch was fitted appropriately by the research team or if further adaptations should be considered.

**Table 3:** Participant responses on watch accuracy across both sessions and responses on watch comfort on the second testing session. S1: session 1 (n = 102) and S2 session 2 (n = 95).

Accuracy (0–100)	S1 M (SD).		S2 M (SD).			
↑HR	64.09 (22.21)		63.48 (24.30)			
↓HR	63.84 (22.46)		61.90 (24.32)			
Comfort	S 2					
	Strongly disagree	Disagree	Neutral	Agree	Strongly Agree	Missing
	1.1%	2.1%	5.3%	24.2%	15.8%	51.6%

### Calming technique practice

Following the first session, participants were encouraged to practice the slow breathing technique until their next session, which occurred 6–8 days later. Practice was not obligatory, and participants’ self-reported practice rates were measured by a questionnaire, to further understand engagement outside of the research setting. Among participants who attended the second session, between group t-test identified no significant differences between the InMe and control arm in practice rates (*t* = −0.91, *df* = 92, *p* = .36, 95% CI [−0.59; −0.22]). However, at follow up, significant differences (*t* = −2.57, *df* = 87, *p* = .01, 95% CI [−0.96; −0.12]) between the InMe (M = 1.33; SD = 1.33) and control (M = 0.70; SD = 0.91) arms were identified. Therefore, evidence suggested that InMe participants were more likely to practice the slow breathing technique introduced in the intervention than the guided imagery technique. In the second session, participants were asked how likely they were to practice the demonstrated calming technique in the 7–10 week period until the follow up session. Half of participants reported that they were likely to practice the technique shown, however at follow up when participants were asked if they did, only 15.73% reported to having practiced more than once. Interestingly, more participants reported practicing between the second and follow up sessions, which could suggest that repeated exposure and familiarisation to the breathing technique, and more time between the sessions, might promote home practice.

### Open ended questions

Following each of the two first session, participants in the InMe group were asked open-ended questions on watch usefulness and prompted to provide examples. Across both sessions InMe participants considered being able to see their heart rate as useful (S1: 22/50; 44.0% and 34/39; S2: 69.39%). Among the majority of first session participants considering the breathing technique as useful, 45.45% (10/22) would use it as a means to relax while sitting an exam or having an assessment or deadline. Following the second session, 17.65% (6/34) of participants would use the technique to relax when running late for a commitment, 14.71% (5/34) while sitting an exam or having an assessment or deadline, and 11.76% (4/34) when feeling overwhelmed from managing multiple responsibilities. Participants considered the breathing exercise useful during the first session and even more so during the second session. This observation underlines that after repeated exposure to the breathing technique, participants understand its usefulness and how it can be applied to their routine. Moreover, given the participant population of university students, it was not surprising that the majority would utilise the slow breathing technique in a relevant high stressful situations of their current lives, such as sitting exams. Future studies should focus on assessing whether clinical populations would utilise heart rate monitoring as a stress relief tool for similar stress-inducing life events.

## Discussion

Recruitment was considered feasible for the present sample and the overall aims of the trial. Overall withdrawal rates did not differ in participants with high self-reported disordered eating behaviours and somatic symptoms. However, many participants with high EDE-Q and PH-Q scores, withdrew prior to attending the first session. This reflects a previous observation of disengagement from the intervention after randomisation, which has become an increasingly significant issue in the recent years [54]. Therefore, greater emphasis should be placed on identifying enablers of participant withdrawal in a specific clinical setting, as motivators in a university setting, differ from clinical settings [55]. Following conversations with experts by experience within the PPI collaboration, clinical populations may be concerned about the type of intervention conducted, the presence of a waiting list, and the ease of accessing the research facility [43]. Therefore, an improved advertisement and recruitment strategy with accessibility information to reach the study site could be an effective step in minimising drop-outs at this stage of the intervention [54].

Additionally, delivering the intervention required three experimenters to be present at the study site, which was not always feasible. All experimenters received thorough training, allowing them to perform multiple roles within the trial. Despite these measures, a substantial portion of physiological data was missing due to technical difficulties. Missing data reduces the statistical power of the analysis, which compromises the overall quality of the data. Especially, in clinical trials where samples are more heterogeneous (i.e., healthy controls and patients) missing data can lead to biased outcomes and poor estimates of the treatment effect of an intervention. Therefore, given the large number of missing data in this trial, future planning should consider appropriate analysis to mitigate this challenge. Specifically, missing data should be considered across different stages of the trial [56]. Additionally, sensitivity analysis should be carried out, which delineates whether missing data are missing at random or whether there are systematic differences between participants with complete data and ones with missing data [56,57].

In terms of intervention feasibility and acceptability, most participants proceeded with the stressors, but a few were not willing to complete either or both stressors, which resulted in disruptions in the study protocol. Although the numbers do not greatly differ, it is evident participants would rather perform the mental math stressor rather than the job or society interview stressor. This could be attributed to the fact that interview stressors might be perceived as more challenging, as they create “a social-evaluative threat”, i.e., a threat of being judged by the panel members [58]. Indeed, more individuals experience anxiety in public speaking [59] rather than mathematics task performance. As a number of participants in this non-clinical sample were reluctant to complete the interview stressor, it is likely that rates of non-compliance may be higher in clinical populations. Accordingly, future research should consider incorporating alternative methods of physiological upregulation (e.g., physical exercise) that elevate heart rate while still enabling participants to engage with the calming technique and maintain closer adherence to the protocol [21]. Overall, participants experienced a greater heart rate increase during the first stressor of each session and a greater decrease following the first slow breathing technique. A diminished heart rate response raises the question of whether the math stressors themselves are inherently less effective in this sample, or if participants exhibited a reduced response due to repetition following the first stressor. A recent review indicates greater variability in responses during the arithmetic task, which may be affect the reduced stress response [58]. Additionally, several participants were considered non-responders due to the decreased stress response following the TSST procedures. This percentage was considered within the normal range and indicates that the adapted TSST procedures were conducted according to protocol and were indeed effective [60]. For the above reasons, clinical populations should have appropriate diagnosis specific stressors. For instance, in the case of EDs, participants could potentially be asked to plan their meals for the weekly schedule (as in the initial clinical audit; [32]), which is stress-inducing but also clinically relevant. Whereas, in the case of somatic symptom disorders, participants could be invited to explain their worst day in terms of symptoms, perform the Hoover’s sign, or the tremor entrainment test [61], which are also considered adequately stressful.

Variability in heart rate reduction was also observed during the calming exercise, which may be attributed to a mismatch between each participant’s optimal relaxation breathing rhythm and the fixed guidance provided by the Polar watch [38]. The watch displayed a slow breathing rhythm of six breaths per minute for all participants, which some participants found challenging, as their optimal rhythm likely differed from the preset pace. Findings also suggest that participants considered the slow breathing exercise more useful, after repeated practice, which points towards the consideration of future clinical studies introducing personalised home practice, as participants were willing to practice in between study sessions. However, it is evident that much less participants reported having practiced the technique than they intended to, as practice between testing sessions was not compulsory. This tendency can be attributed to what is known as the intention to action gap, in which is the discrepancy between the intention to act and the realisation of behaviour [62]. It is suggested that perceived self-efficacy beliefs, the belief in the ability to perform a given task, can contribute to the realisation of the intended action [63,64]. Therefore, if this intervention can strengthen self-efficacy beliefs, participants may be better able to bridge the gap between intention and action. Given that participants reported difficulty implementing the breathing technique but nevertheless expressed willingness to engage in practice outside the session, future studies may benefit from incorporating monitored home-based practice between research visits. This suggestion could be realised through mobile apps [65,66], where participants can follow slow breathing visualisations and log their practice time. Furthermore, instruction in the breathing technique should extend beyond watch based delivery. Incorporating in-person guidance from a trained experimenter may enhance participant comprehension and adherence.

### Future considerations

Although a third of participants found the slow breathing technique challenging to follow, they were willing to engage in home practice; therefore, incorporating home practice between testing sessions is recommended. This approach would allow participants to become more familiar with slow breathing, making it easier for them to regulate their heart rate during stressful situations. Additionally, tailoring the breathing technique to each participant’s optimal rhythm should be considered as a future improvement. However, research exploring technologies is still in the pilot phase [67].

For clinical populations, additional improvements should be considered. As aforementioned, motivators to participate for clinical populations may differ significantly from those of university students, it is important to consider how best to approach clinical populations. Therefore, providing detailed accessibility information to the study site particularly for individuals with somatic symptom disorders, might reduce ambiguity and stress associated with access to the study site. Similarly for patients with EDs a detailed description of the intervention would also reduce stress associated with the intervention. Additionally, incorporating flexibility in testing schedules is strongly recommended, as the preparation and administration of stressor-based procedures in clinical populations may require extended or variable testing durations. Lastly, using diagnosis-specific stressors tailored to the clinical sample being recruited is strongly recommended due to the decreased heart rate response after repeated stressor exposure. Suggestion for future interventions and clinical populations specifically are shown in Box 1 below.

**Box 1. Considerations for future interventions.**

**Non-clinical populations**

- Personalised slow breathing rhythm
- Calming technique practice can be monitored via mobile phone app

**Clinical populations**

- Access details to the research site (i.e., wheelchair accessibility, map printout, clear instructions)
- Adequate information available about the intervention
- Flexibility in testing schedules
- Stressors relevant to clinical sample (e.g., meal planning, Hoover’s sign, tremor entrainment test)

## Conclusion

In conclusion, this feasibility and acceptability study revealed that the InMe intervention was feasible in terms of recruitment procedures, intervention procedures, including the use of a smartwatch, a slow breathing procedure, and different stressor procedures and the trial’s multiple measurement tools, ranging from qualitative to quantitative measures. The trial was also largely accepted by trial participants, despite the complexity of the involved stressor procedures and the noted difficulty of the slow breathing procedure. To our knowledge, this is the first study assessing the feasibility and acceptability outcomes of an RCT that targets interoception at a behavioural level and a physiological level (i.e., fluctuation of heart rate). Our conclusions hold potential in informing the feasibility and acceptability of future interventions implemented either in subclinical settings (e.g., universities) or translated into clinical settings.

## Supporting information

S1 Table Guided imagery technique for control arm participants. Participants assigned to the active control arm had the above script read to them by the researcher leading the testing session. The researcher ensures the script is read within 3 minutes, which allows enough time for participants to downregulate their stress response.
(DOCX)

S2 Table Heart rate calculation formulas for TSST upregulation and slow breathing technique downregulation. (DOCX)

S3 Table Demographic information of gender, disordered eating behaviours (EDE-Q) and somatic symptoms (PHQ-15) in session 1 (n = 102). (DOCX)

S4 Table Mean duration of each testing session with standard deviation in both arms and in total. (DOCX)
