# Supplementary material for: Feasibility and acceptability outcomes of the InMe trial - a randomised controlled trial in participants with subclinical eating and somatic symptom disorders
Source: PLoS One. 2026 Feb 4;21(2):e0342307. doi: 10.1371/journal.pone.0342307 (PMC12871983; doi:10.1371/journal.pone.0342307)
Supplement: S1 Table — Participants assigned to the active control arm had the above script read to them by the researcher leading the testing session. The researcher ensures the script is read within 3 minutes, which allows enough time for participants to downregulate their stress response. (DOCX) [file pone.0342307.s001.docx]

**S1 Table.** **Guided imagery technique for control arm participants.** Participants assigned to the active control arm had the above script read to them by the researcher leading the testing session. The researcher ensures the script is read within 3 minutes, which allows enough time for participants to downregulate their stress response.

Imagine a place where you feel calm and peaceful and easy.... a place either make believe or real.... a place from your past.... or somewhere you’ve always wanted to go.... It doesn’t matter which place, as long as it’s a place you feel safe and comfortable. Use your imagination to picture yourself walking slowly. Look around, whatever is pleasing to you can be found in this place.... Perhaps there are flowers, trees, animals, birds, water, or even music...Perhaps there are other people that you invited to this safe and calm place. Look around… feel the air on your skin, notice the temperature, notice the air… listen to the sounds of the place... smell the different fragrances... can you smell one or more fragrances… imagine the texture of the ground beneath your feet.... is it soft? Spend some time exploring, using your sense of touch as you continue to feel at peace and comfortable. Know that you can come back to this place... whenever you wish... to help your mind relax and imagining yourself in your safe place.  Keeping your eyes closed for just a little longer... taking a moment to notice how you are feeling... When you are ready to leave.  As you bring yourself to the here and now, remember that you can use your imagination to return to your safe place at any time you wish. Slowly come back into the room... open your eyes whenever you are ready. Stretch gently and open your eyes, feeling refreshed and alert”.
