# Supplementary material for: Feasibility and acceptability outcomes of the InMe trial - a randomised controlled trial in participants with subclinical eating and somatic symptom disorders
Source: PLoS One. 2026 Feb 4;21(2):e0342307. doi: 10.1371/journal.pone.0342307 (PMC12871983; doi:10.1371/journal.pone.0342307)
Supplement: S2 Table — (DOCX) [file pone.0342307.s002.docx]

**S2 Table. Heart rate calculation formulas for TSST upregulation and slow breathing technique downregulation.**

| **Stressor** | **Heart rate** | **Calculation Formulas** |
| --- | --- | --- |
| Job / Society | Upregulation | Before given task instructions - In anticipation of the job or society stressor. |
| Math | Downregulation | Post society / job speech - Post breathing technique. |
| Job / Society | Upregulation | Post breathing technique - Prior math stressor. |
| Math | Downregulation | In anticipation of the math stressor - Post breathing technique. |
