# Supplementary material for: Feasibility and acceptability outcomes of the InMe trial - a randomised controlled trial in participants with subclinical eating and somatic symptom disorders
Source: PLoS One. 2026 Feb 4;21(2):e0342307. doi: 10.1371/journal.pone.0342307 (PMC12871983; doi:10.1371/journal.pone.0342307)
Supplement: S3 Table — (DOCX) [file pone.0342307.s003.docx]

**S3 Table,** **Demographic information of gender, disordered eating behaviours (EDE-Q) and somatic symptoms (PHQ-15) in session 1 (n= 102).**

|  | EDE-Q | | | | PHQ-15 | | | |
| --- | --- | --- | --- | --- | --- | --- | --- | --- |
|  | InMe | | Control | | InMe | | Control | |
|  | High | Low | High | Low | High | Low | High | Low |
| Female | 9 | 32 | 10 | 33 | 4 | 37 | 8 | 35 |
| Male | 2 | 5 | 0 | 8 | 0 | 7 | 1 | 7 |
| Non - Binary | 0 | 2 | 0 | 1 | 1 | 1 | 0 | 1 |
| Total | 11 | 39 | 10 | 42 | 5 | 45 | 9 | 43 |
