# Supplementary material for: Feasibility and acceptability outcomes of the InMe trial - a randomised controlled trial in participants with subclinical eating and somatic symptom disorders
Source: PLoS One. 2026 Feb 4;21(2):e0342307. doi: 10.1371/journal.pone.0342307 (PMC12871983; doi:10.1371/journal.pone.0342307)
Supplement: S4 Table — (DOCX) [file pone.0342307.s004.docx]

**S4 Table.** M**ean duration of each testing session with standard deviation in both arms and in total.**

|  | **InMe arm** | **Control arm** | **Total** |
| --- | --- | --- | --- |
| Session 1 | 1 hour 47 min (14) | 1 hour 29 min (13 min) | 1 hour 38 min (16 min) |
| Session 2 | 1 hour 43 min (10 min) | 1 hour 32 min (9 min) | 1 hour 38 min (11 min) |
| Follow up | 1 hour 1 min (10 min) | 60 min (14 min) | 1 hour 1 min (12 min) |
